# Characteristics of Emergency Visits Among Lung Cancer Patients in Comprehensive Cancer Center and Impact of Palliative Referral

**DOI:** 10.7759/cureus.37903

**Published:** 2023-04-20

**Authors:** Munzir M Alandonisi, Hussain J Al-Malki, Waleed Bahaj, Hosam A Alghanmi

**Affiliations:** 1 Department of Medical Oncology, Oncology Center, King Abdullah Medical City, Makkah, SAU; 2 Department of Medical Oncology, Armed Forces Hospital South Region, Khamis Mushait, SAU

**Keywords:** characteristics, impact, palliative care, emergency visits, lung cancer

## Abstract

Introduction: During the treatment course, cancer patients are prone to develop acute symptoms that are either treatment-related or cancer-related. Emergency services are available during the whole day to manage the acute problems of patients with chronic diseases, including cancer patients. Previous studies have shown that palliative care (PC) provided at the beginning of stage IV lung cancer diagnosis helped to reduce emergency visits and increase survival rates.

Method: A retrospective study was conducted on lung cancer patients with confirmed histopathology of non-small cell cancer and small cell lung cancer who visited the emergency department (ED) from 2019 to 2021. The demographic data, disease-related-data causes of ED visits (including disposition), number of emergency visits, and palliative referral and impact on the outcome and frequency of emergency visits were reviewed.

Results: Of a total number of 107 patients, the majority were male (68%), the median age was 64 years old, and almost half of them were smokers (51%). More than 90% of the patients were diagnosed with non-small cell lung cancer (NSCLC), more than 90% with stage IV, and a minority underwent surgery and radiation therapy. The total number of ED visits amounted to 256, and 70% of the reasons for ED visits were respiratory problems (36.57%), pain (19.4%), and gastrointestinal (GI) causes (19%), respectively. PC referral was performed only for 36% of the participants, but it had no impact on the frequency of ED visits (p-value > 0.05). Besides, the frequency of ED visits had no impact on the outcome (p-value > 0.05), whereas PC had an impact on the live status (p-value < 0.05).

Conclusion: Our study had similar findings to another study regarding the most common reason for ED visits among lung cancer patients. Improving PC engagement for patient care would render those reasons preventable and affordable. The palliative referral improved survival among our participants but had no impact on the frequency of emergency visits, which may be due to the small number of patients and the different populations included in our research. A national study should be conducted to obtain a larger sample and to determine the impact of PC on ED visits.

## Introduction

Cancer is considered the leading cause of death and a significant impediment to extending life expectancy. In regard to global cancer statics, lung cancers are the top cause of death in male cancer-related death and the second cause of death among females after breast cancer [1]. With advances in the treatment of cancer patients, life expectancy has increased and the treatment of related cancer symptoms would increase with time. It is inevitable for oncology patients to visit the emergency department (ED) during their treatment journey [2]. The ED is an essential service that provides healthcare for any patients with acute symptoms [3].

Perhaps in part because of the increasing number of cancer survivors, studies have shown that cancer patients are increasingly likely to visit an ED in general hospitals and cancer centers [4,5]. Cancer patients usually require emergency medical care due to acute unbearable symptoms and life-threatening conditions. The employment of EDs by such patients can negatively affect both the individuals and the ED system [6]. Visits to the emergency room (ER), admission to the hospital from the ER, and death in the ER are more frequent among lung cancer patients than those with other types of cancer [7].

However, in order to reduce unnecessary ED visits among this population, more studies are required about the characteristics of cancer-related ED visits, which may as well play a critical role in improving cancer care delivery.

According to a study from the United States, lung cancer is the most common cancer among patients visiting the ED [8]. The study also showed that the main reason for ED visits was pain, respiratory problems, and neurological symptoms. Other studies that were conducted in Japan reported that frequent ED visits have a negative drawback on the outcome of lung cancer [7].

The integration of a palliative care (PC) team in the treatment of cancer patients has a positive impact on the outcome, quality of life, and symptoms of such patients and caregivers [9].

Further research on cancer-related ED visits plays a significant role in improving the quality of cancer treatment since the number of unnecessary ED visits could be reduced if the clinical needs of a cancer patient are understood. The purpose of this study is primarily to provide a comprehensive assessment of the characteristics and chief complaints of ED visits for lung cancer patients and secondarily to assess the PC referral in early cancer diagnosis and the impact of ED visits on lung cancer patients.

## Materials and methods

Study design

A retrospective study was conducted among people with lung cancer who visited an ED from 2019 to 2021. We analyzed ED visits from our specialized electronic health system.

Study population

A generated list of all patients labeled as lung cancer patients (small cell lung cancer (SCLC) and non-small cell lung cancer (NSCLC) in our cancer center) was obtained from the electronic record. Patients who met the inclusion criteria were asked to complete a data sheet as a retrospective-based questionnaire prepared by the investigators.

Inclusion and exclusion criteria

In our retrospective study, patients who were confirmed to have a histopathological diagnosis of lung cancer, including SCLC and NSCLC, and who started active oncology treatment were included. Patients with different histopathologies, such as mesothelioma and neuroendocrine tumor among others, or patients who did not receive active oncology treatment were excluded.

Statistical analysis

A descriptive analysis was conducted to determine the disease status (stage, mutation, and treatment received during the study). The frequency and percentage of emergency visits, causes of emergency visits, and disposition after the emergency visits were calculated. All analyses were completed by using Microsoft Excel version 2021, and the statistical significance was regarded as a 2-sided P value.

## Results

Of a total of 107 patients included, the majority were male (68%), the median age was 64 years old, and more than half of them were smokers (50.4%) (Table 1).

**Table 1:** Demographic data

Demographic	
Gender (n,%)	
Female	(33) 31.78%
Male	(74) 68.22%
Grand Total	(107) 100.00%
Age Mean, median, and SD	
Mean	63.70
Median	64
Standard Deviation	11.28
Smoking status (n,%)	
No	(52) 49.53%
Yes	(55) 50.47%
Grand Total	(107) 100.00%

The majority were NSCLC patients (more than 90%) with adenocarcinoma (73.83%), squamous cell carcinoma (14.95%), and adenosquamous (2.8%), respectively. Upon the emergency visits, most of the participants were stage IV cancer patients (more than 75%). A mutation analysis of the participants showed that one-third of them were PDL expression (33.33%) and the rest were EGFR mutant (43.59%), ALK mutant (15.38%), and ROS-1 mutant (7.69%). A minority of the patients underwent surgery (7.48%) and (34.58%) received radiation therapy. The palliative referral was performed in one-third of the patients (36.45%). About half of the participants were alive during the study (49.6%) and were on active cancer treatment (45.45%) (Table 2).

**Table 2:** Disease-related data SCLC: small cell lung cancer

Disease-related data	
Histopathology	(n,%)
Adenocarcinoma	(78) 73.83%
Adenosqeoumus	(4) 2.80%
Large cell carcinoma	(3) 1.87%
SCLC	(7) 6.54%
Squamous cell carcinoma	(15) 14.95%
Grand Total	(107) 100.00%
Stage upon emergency visit	(n,%)
Extensive stage (SCLC)	(5) 4.67%
Limited stage (SCLC)	(2) 0.93%
Stage I	(4) 3.74%
Stage II	(4) 3.74%
Stage III	(12) 11.21%
Stage IV	(80) 75.70%
Grand Total	(107) 100.00%
Mutation Status	(n,%)
ALK mutant	(12) 15.38%
EGFR mutant	(34) 43.59%
PDL-1 expression	(27) 33.33%
ROS-1 mutant	(7) 7.69%
Grand Total	(80) 100.00%
Surgery	(n,%)
No	(99) 92.52%
Yes	(8) 7.48%
Grand Total	(107) 100.00%
Type of surgery	(n,%)
Lobectomy	(7) 87.50%
Pneumonectomy	(1) 12.50%
Grand Total	(8) 100.00%
Received Radiation	(n,%)
No	(69) 65.42%
Yes	(38) 34.58%
Grand Total	(107) 100.00%
Palliative referral	(n,%)
No	(68) 63.55%
Yes	(39) 36.45%
Grand Total	(107) 100.00%
On cancer therapy during the study	(n,%)
No	(58) 54.55%
Yes	(49) 45.45%
Grand Total	(107) 100.00%
Patient still alive	(n,%)
No	(54) 50.4%
Yes	(53) 49.6%
Grand Total	(107) 100.00%

A total of 28 patients had no emergency visits. A total of 79 patients had one visit and 54 of them had two visits. Several patients had between three and four visits (36 had three visits and 28 had four visits). Furthermore, 18 patients had more than four visits (Table 3).

**Table 3:** Number of emergency visits

Number of emergency visits	
No emergency visit	28 patients
1-2 emergency visits	133 visits (79 patients had one visit and 54 had two visits)
3-4 emergency visits	64 visits (36 patients had three visits and 28 patients had four visits)
More than 4 emergency visits	59 visits (18 patients)

The total number of emergency visits was 256, and around 40% of these patients were admitted. Afterward, emergency dispositions were made to regular rooms (73%), isolation rooms (59%), and intensive care units (4%), respectively. The majority of ED visits' causes were respiratory problems (36%), pain (19%), and gastrointestinal (GI) symptoms (19%) followed by other causes like dehydration, neurological issues, and fatigue (Table 4).

**Table 4:** Disposition after emergency visits and causes of visits

Items	(n, %)
Total emergency visits	(256) 100%
Required admission (n,%)	
Yes	(101) 39.68%
No	(155) 60.30%
If required admission, disposition (n,%)	
Intensive Care Unite	(4) 4.40%
Isolation room	(21) 21.30%
Regular room	(76) 74.30%
Causes of emergency visit (n,%)	
Dehydration	(10) 3.89%
Fatigue	(20) 7.78%
Fever (infection)	(20) 7.78%
Gastrointestinal symptoms	(49) 19.06%
Injury	(1) 0.38%
Neurological symptoms	(13) 5.05%
Pain	(50) 19.45%
Respiratory symptoms	(94) 36.57%

The frequency of emergency visits and their impact are shown in Table 5. The frequency of ER visits does not impact the live status, and the P-value was found to be > 0.05.

**Table 5:** Correlation of death rate to the frequency of emergency visits

Emergency visits	P value
No visit	0.050
1-2 visits	0.562
3-4 visits	0.198
More than 4 visits	0.615

A palliative referral does not impact the frequency of ER visits and the P-value (> 0.05) as shown in Table 6. The palliative referral is found to have a positive impact on the live status with a P-value of less than 0.05.

**Table 6:** Impact of palliative referral on the frequency of ED visits and life status ED: emergency department

Emergency visits	P value
No visit	0.721
1-2 visits	0.914
3-4 visits	0.196
More than 4 visits	0.627
Palliative referral and impact on life status (dead/alive)	<0.005

## Discussion

Visits to the ER, admission to the hospital from the ER, and death in the ER are more frequent among patients with lung cancer than patients with other types of cancer [7]. Moreover, PC is one of the most comprehensive oncologic services needed for advanced oncologic cases to reduce the disease burden and use of health services and to improve patient’s quality of life [10]. The randomized controlled trial involving PC at the time of stage IV lung cancer diagnosis showed an improved overall survival rate compared to standard oncologic treatment only [11].

In this retrospective study, the majority of participants were stage IV NSCLC (75%) and half of the patients were alive during the timeline of data collection. Moreover, less than half of the patients required admission upon ED visits. The top three reasons for ER visits were respiratory problems, pain, and GI symptoms. Our study found that palliative referrals improve survival among patients. Unfortunately, only one-third of the participants (36.4%) had palliative referrals in our research compared to almost 70% in a study conducted in the United States of America (either early within three months of diagnosis or after) [9].

In the study conducted in the United States of America, the palliative referral was associated with either early palliative referral or outpatient referral and fewer ER visits (39% vs. 68%, P < 0.001), hospitalizations (48% vs. 81%, P < 0.003), and hospital deaths (17% vs. 31%, P = 0.004). Similarly, an outpatient palliative referral was associated with fewer ER visits (48% vs. 68%, P < 0.001), hospital admissions (52% vs. 86%, P < 0.001), hospital deaths (18% vs. 34%, P = 0.001), and intensive care unit admissions (4% vs. 14%, P = 0.001). According to a Korean study, the frequency of ER visits among lung cancer patients predicts hospitalization and death [12]. Our study found that palliative referral improved survival among our participants but had no impact on the frequency of emergency visits even though the frequency of emergency visits had no impact on the live status. These findings are not in line with previous findings in regard to the association between frequent ER visits and fewer ER visits with PC. This could be due to the small number of patients and the different populations included in our research. Therefore, a national study should be performed.

Commonly encountered reasons for ER visits were respiratory issues (36%), pain (19%), and GI symptoms (19%), respectively, followed by fever (7%), fatigue (7%), and neurological symptoms (5%) respectively. A Korean study also reported respiratory causes (36%) followed by pain (12%) [12], and another report from Japan confirmed that the most encountered ER causes were respiratory problems, pain, and GI symptoms, respectively [7]. Similarly, studies conducted to determine the potentially preventable ER visits among cancer patients found that between 41.3% and 63.5% of the causes could be prevented (14.4% were lung cancer cases) [2]. Our study claimed that determining the preventable causes and enhancing management as an outpatient would help to reduce ER visits and unnecessary additional costs [2]. Based on our findings and other studies, most of the encountered causes could be prevented through the integration of PC and could decrease the burden on the health services and patients of waiting a long time to receive ER management for preventable causes. Our study had certain limitations; it was center-based and involved only a small number of patients. Hence, we should conduct a study with a greater number of participants to determine the impact of PC from the start in lung cancer patients and to enhance medical oncologist referral as soon as the patient is diagnosed.

## Conclusions

Lung cancer patients are more likely to visit ED than patients with other types of cancer. The most common causes of ED visits were respiratory troubles, pain, and GI problems. Furthermore, most of those causes could have been avoided if PC was involved. Since frequent emergency visits significantly burden the ED, the implementation of a clear pathway for such patients would improve their quality of life and symptoms and decrease the health services’ burden.
